# Supplementary material for: Uncovering COVID-19 transmission tree: identifying traced and untraced infections in an infection network
Source: Front Public Health. 2024 Jun 3;12:1362823. doi: 10.3389/fpubh.2024.1362823 (PMC11180726; doi:10.3389/fpubh.2024.1362823)
Supplement: Supplementary file 1 [file Data_Sheet_1.pdf]

APPENDIX

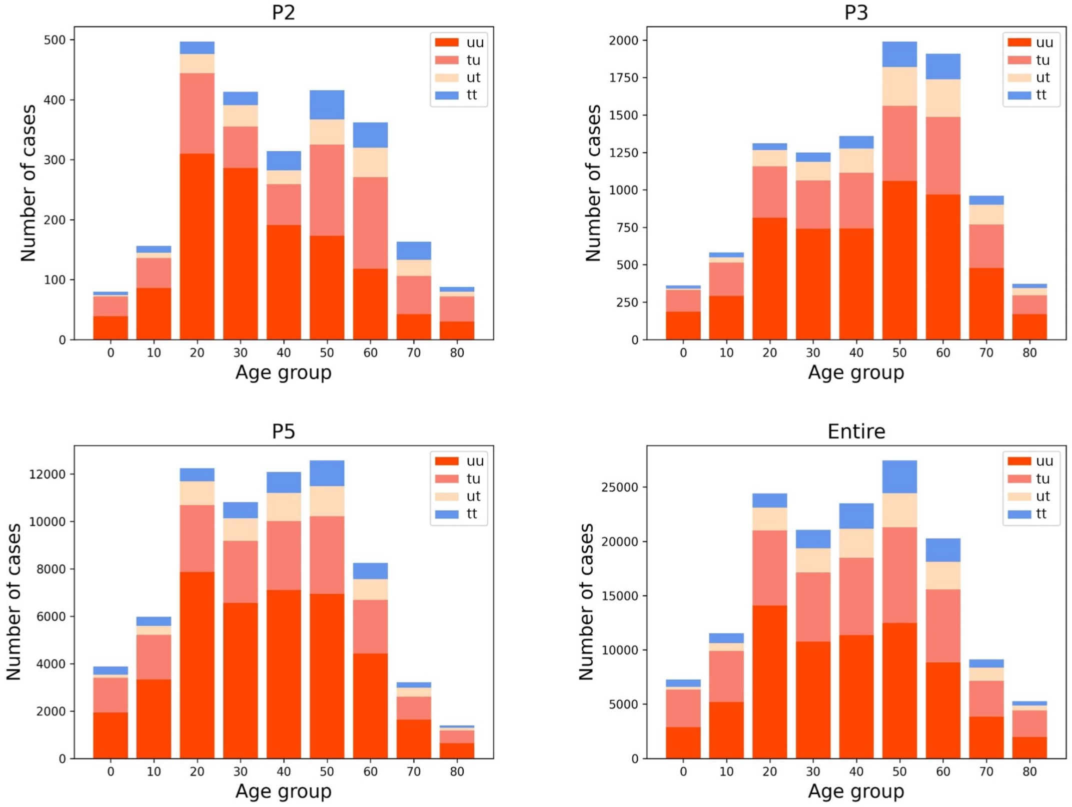

Figure A1. Additional information for the upper panel of Figure 3. Age distribution categorized according to four types for both $P2$, $P3$, $P5$ and Entire period.

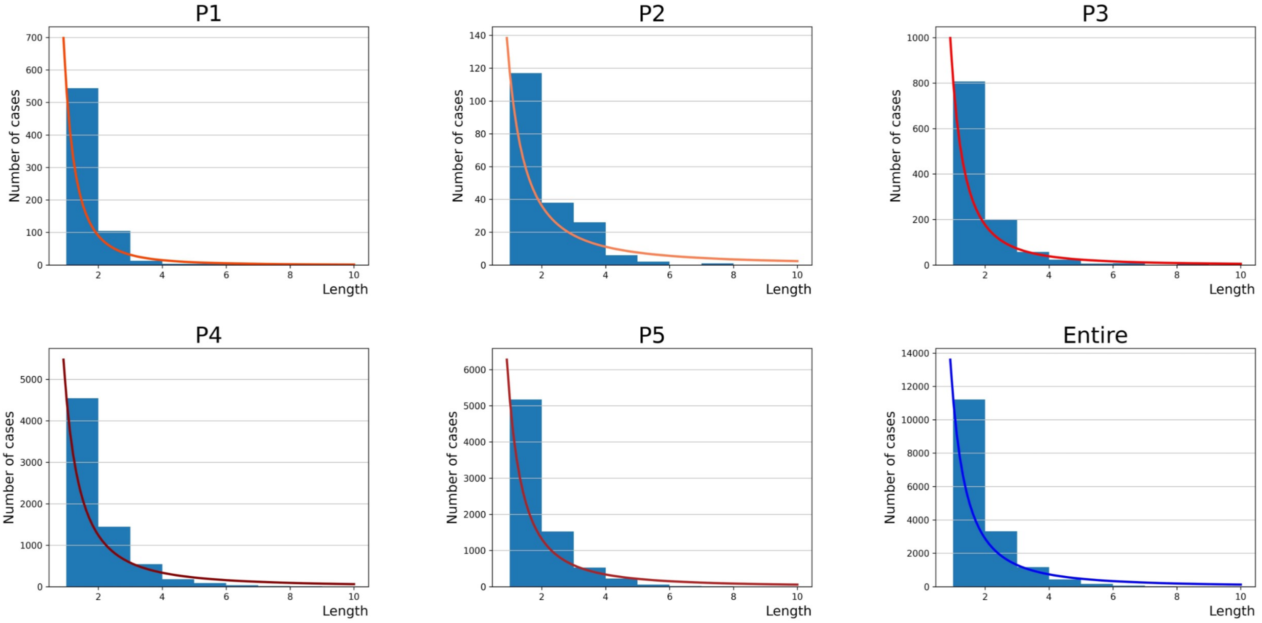

Figure A2. Additional information for the upper panel of Figure 5. The figure presents the distribution of connected component length for each period.
